# Liver blood dynamics after bariatric surgery: the effects of mixed-meal test and incretin infusions

**DOI:** 10.1530/EC-18-0234

**Published:** 2018-06-25

**Authors:** Henri Honka, Jukka Koffert, Saila Kauhanen, Nobuyuki Kudomi, Saija Hurme, Andrea Mari, Andreas Lindqvist, Nils Wierup, Riitta Parkkola, Leif Groop, Pirjo Nuutila

**Affiliations:** 1 Turku PET CentreUniversity of Turku, Turku, Finland; 2 Department of GastroenterologyTurku University Hospital, Turku, Finland; 3 Division of Digestive Surgery and UrologyTurku University Hospital, Turku, Finland; 4 Faculty of MedicineKagawa University, Kagawa, Japan; 5 Department of BiostatisticsUniversity of Turku, Turku, Finland; 6 Institute of NeuroscienceNational Research Council, Padua, Italy; 7 Department of Clinical SciencesLund University Diabetes Centre, Malmö, Sweden; 8 Department of RadiologyUniversity of Turku and Turku University Hospital, Turku, Finland; 9 Department of EndocrinologyTurku University Hospital, Turku, Finland

**Keywords:** portal vein blood flow, hepatic blood volume, glucose-dependent insulinotropic polypeptide, bariatric surgery, positron emission tomography

## Abstract

**Aims/hypothesis:**

The mechanisms for improved glycemic control after bariatric surgery in subjects with type 2 diabetes (T2D) are not fully known. We hypothesized that dynamic hepatic blood responses to a mixed-meal are changed after bariatric surgery in parallel with an improvement in glucose tolerance.

**Methods:**

A total of ten morbidly obese subjects with T2D were recruited to receive a mixed-meal and a glucose-dependent insulinotropic polypeptide (GIP) infusion before and early after (within a median of less than three months) bariatric surgery, and hepatic blood flow and volume (HBV) were measured repeatedly with combined positron emission tomography/MRI. Ten lean non-diabetic individuals served as controls.

**Results:**

Bariatric surgery leads to a significant decrease in weight, accompanied with an improved β-cell function and glucagon-like peptide 1 (GLP-1) secretion, and a reduction in liver volume. Blood flow in portal vein (PV) was increased by 1.65-fold (*P* = 0.026) in response to a mixed-meal in subjects after surgery, while HBV decreased in all groups (*P* < 0.001). When the effect of GIP infusion was tested separately, no change in hepatic arterial and PV flow was observed, but HBV decreased as seen during the mixed-meal test.

**Conclusions/interpretation:**

Early after bariatric surgery, PV flow response to a mixed-meal is augmented, improving digestion and nutrient absorption. GIP influences the post-prandial reduction in HBV thereby diverting blood to the extrahepatic sites.

## Introduction

The liver plays a pivotal role in the regulation of human glucose metabolism, and defects in hepatic insulin signaling predispose to hyperglycemia and type 2 diabetes (T2D) (1, 2). Portal delivery of ingested glucose and other nutrients and hormones secreted from the gut and pancreas elicit a shift in hepatic glucose metabolism toward net uptake (3). However, little is known about hepatic blood flow and volume (HBV) responses to a mixed-meal after bariatric surgery.

The incretin hormones glucose-dependent insulinotropic polypeptide (GIP) and glucagon-like peptide 1 (GLP-1) account for the gut-derived amplification of insulin secretion (4). In addition to the effect on pancreatic islets, we (5) and others (6) have shown that GIP contributes to the redistribution of gastrointestinal blood flow after meal ingestion. Whether these extrahepatic splanchnic vascular effects are reflected by changes in hepatic blood dynamics is not known.

To address this, we quantitated blood flow in portal vein (PV) and hepatic artery (HA), and HBV during a mixed-meal test and GIP infusion in morbidly obese subjects with T2D and age-matched lean controls with positron emission tomography/magnetic resonance imaging (PET/MRI). The experiments were repeated in subjects early after bariatric surgery to clarify the effects of altered gastrointestinal anatomy rather than that of fully established weight loss on glucose tolerance and hepatic blood dynamics.

## Materials and methods

### Participants

Ten morbidly obese non-smoking subjects with T2D (age = 47 (interquartile range 46–59) years; weight = 121 (95.3–130) kg; HbA1c = 40.5 (37.8–42.8) mmol/mol) participated in the study. A total of nine subjects received antidiabetic therapy, whereas one subject was managed by diet only. In addition, ten lean non-diabetic controls (age = 50 (46–52) years, *P* = 0.569) were recruited. The study protocol was approved by the Ethical Committee of the Hospital District of Southwestern Finland (ClinicalTrials.gov Identifier Nbib1880827) and written informed consent was obtained from all participants prior to enrollment.

### Study design

Study design and PET experimentation have previously been described (7). During the screening visit, a 2-h oral glucose tolerance test (OGTT) was performed on all participants, and the diagnosis of diabetes was confirmed in obese subjects. Thereafter, each participant underwent a mixed-meal testing and GIP infusion followed by a PET acquisition on two separate days (8). Controls were also studied during GLP-1 infusion on a third day. Subjects had a drug wash-out period (72 h for metformin and dipeptidyl peptidase IV inhibitors, 10 weeks for GLP-1 receptor agonists and 24 h for antihypertensives) prior to the experiments.

**Figure 1:**
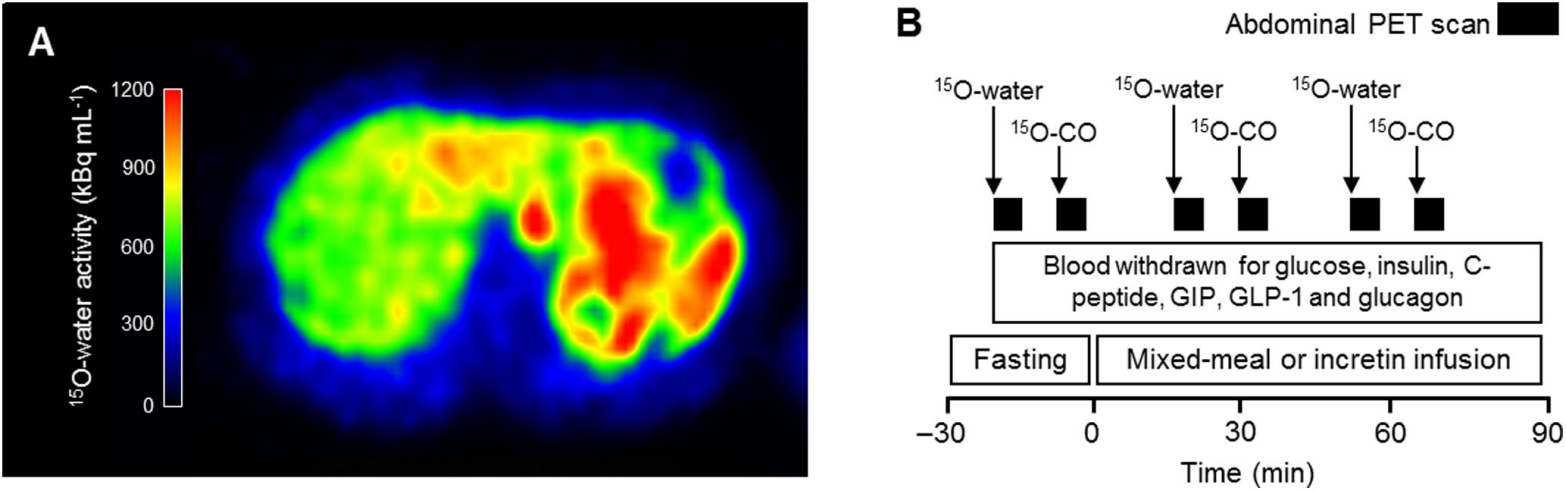
An example of an abdominal ^15^O-water PET image (A) during the mixed-meal test in lean non-diabetic control. Experimental study design (B). Arrows indicate radiotracer administration and black bars abdominal PET scan, respectively. ^15^O-CO, ^15^O-carbon monoxide.

Participants reported to the Turku PET Centre after an overnight fast. Peripheral catheters were placed in both cubital veins, one for blood sampling and other for radiotracer and incretin administration. Splanchnic blood flow and volume were measured with positron emitting ^15^O-water and ^15^O-carbon monoxide, respectively. The former is a freely-diffusible tracer used to evaluate blood flow through tissues, while the latter tracer is bound to hemoglobin with high affinity. After the whole-body T_2_-weighted MR imaging, baseline ^15^O-water (median dose 483 (467–507) mBq, IV injection) and ^15^O-carbon monoxide (median dose 769 (699–800) MBq, inhalation) PET scans of the abdomen were obtained (Fig. 1A and B). During the experiments, blood was sampled at time points 0, 15, 30, 45, 60 and 90 min to measure glucose, insulin, C-peptide, glucagon, total GIP and active GLP-1.

### Bariatric surgery

Surgical procedures (either Roux-en-Y gastric bypass (RYGB) or vertical sleeve gastrectomy (VSG), both *n* = 5) were performed after a very-low-calorie diet, as previously described (9). Mixed-meal testing and GIP infusion were repeated 69 (55–97) and 80 (47–92) days after the surgery, respectively.

### Mixed-meal test

Participants ingested a 250 kcal liquid meal solution (Nutridrink, Nutricia Advanced Medical Nutrition, Amsterdam, Netherlands), consisting of 40 g of carbohydrates, 6 g of fat and 9 g protein, in 10 min. PET scans were repeated twice post ingestion: at 20 and 50 min for ^15^O-water, and at 40 and 70 min for ^15^O-carbon monoxide, respectively. These time points were chosen to cover accelerated gastric emptying rate, intestinal transit and peak GIP and GLP-1 concentrations in the post-bariatric state.

### GIP and GLP-1 infusions

Incretins were supplied by Bachem Holding AG (Bubendorf, Switzerland), and prepared in the hospital pharmacy in sterilized water added with 2% human serum albumin. GIP infusion was primed at the rate of 4.0 pmol/kg/min and after 15 min the rate was halved, as previously described (10). On a separate day, controls received GLP-1 infusion at the constant rate of 0.75 pmol/kg/min. PET scans were repeated twice after the start of the infusions: at 20 and 50 min for ^15^O-water, and at 40 and 70 min for ^15^O-carbon monoxide, respectively, followed by the discontinuation of the infusions.

### PET image processing

Raw data were corrected for dead time, decay and tissue attenuation with MR-based attenuation correction (MRAC) and reconstructed in a 144 × 144 matrix. Hepatic regions-of-interests (ROI) were extracted manually using GPETView (courtesy of Prof. Hiroshi Watabe, downloadable at http://www.rim.cyric.tohoku.ac.jp/software/gpetview/gpetview.html) and Carimas 2 (Turku PET Centre, downloadable at http://turkupetcentre.fi) to obtain dynamic ^15^O-water time-activity curves and static ^15^O-carbon monoxide data.

### Biochemical analyses and hormone assays

Plasma glucose was measured with the glucose oxidase method using a GM9 Analyzer (Analox Instruments, London, UK), and plasma insulin and C-peptide were determined by the immunochemiluminescent assays in the hospital laboratory. Plasma total GIP and active GLP-1 concentrations were measured with an ELISA kit (EMD Millipore, St. Charles, MO, USA). Glucagon was measured using a radioimmunoassay from EMD Millipore.

### Mathematical modeling

PV and HA flow were estimated using a model dual-input functions, as previously described (11). The quantitation of HBV was performed using the following formula:


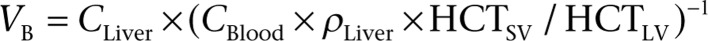


where *V*_B_ is the blood volume in liver tissue (in mL/g), *C*_Liver_ and *C*_Blood_ are radioactivity in liver ROI and abdominal aorta ROI (in Bq/mL), respectively, *ρ*_Liver_ is liver volumetric density of 1.04 g/mL, and HCT_SV_/HCT_LV_ represent the small-to-large vessel hematocrit ratio of 0.85 (12, 13). Liver volumes were manually determined and used to normalize hepatic blood perfusion and volume rates. Insulin sensitivity index (2-h OGIS) and model parameters of β-cell function were calculated from the OGTT-based data as previously described (14, 15). The C-peptide deconvolution method (16) was used to estimate insulin secretion rate (ISR) during the mixed-meal test and infusions. Insulin clearance was calculated as the ratio of ISR-to-insulin concentration.

### Statistical analysis

Variables have been described using medians with interquartile range (IQR). Changes over time and between groups were analyzed using repeated-measurements ANOVA, and the Tukey–Kramer’s method was used to adjust the *P* values of pairwise comparisons. The normality of the residuals was checked for justification of the analyses and transformations were used for non-normally distributed variables. Pearson’s correlation coefficient was calculated to explore the correlations between variables. Two-sided *P* < 0.05 was considered statistically significant. Statistical analyses were performed using SAS System for Windows, version 9.4 (SAS Institute, Cary, NC, USA).

## Results

### Subject characteristics

**Figure 2:**
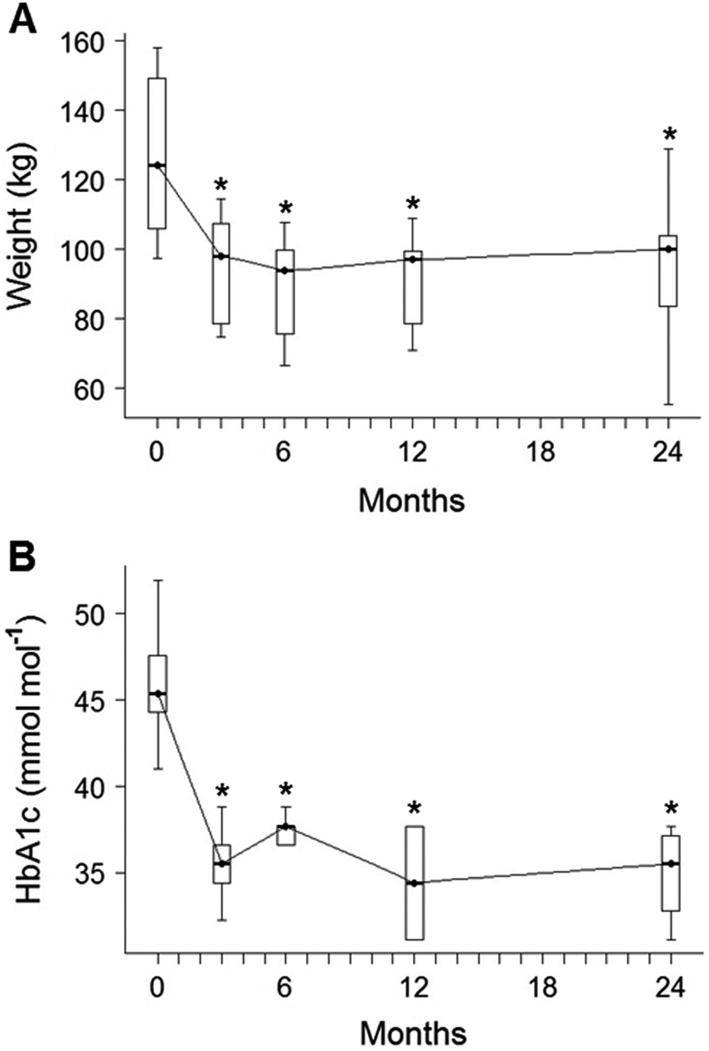
Box-plots of study subjects weight (A) and HbA1c (B) during the 2-year follow-up. *P < 0.05 vs baseline in linear mixed model with Tukey–Kramer’s correction. HbA1c, glycated hemoglobin.

Detailed anthropometric and biochemical data are shown in Table 1. Before surgery, obese subjects were insulin-resistant and hyperglycemic, had higher liver volumes and lower rates of insulin clearance when compared with controls. Early after surgery weight was decreased by a median of 14.7 (12.2–20.4) kg, accompanied with a significant reduction in liver volume and improvements in glucose sensitivity, insulin clearance and sensitivity, and glycemic control, with no difference between RYGB and VSG groups (Supplementary Table 1, see section on supplementary data given at the end of this article). After surgery, five subjects had normal glucose tolerance and only two subjects still needed oral antidiabetic medication. No further reductions in weight and glycated hemoglobin (HbA1c) (Fig. 2A and B) were observed during the 2-year follow-up.

**Table 1:** Participant characteristics.

	Controls	Obese		P*	*P*^†^	*P*^‡^
		Pre-surgery	Post-surgery			
*n* (male/female)	10 (2/8)	10 (2/8)				
Weight (kg)	61.5 (59.3–66.5)	121 (95.3–130)	103 (81–111)	<0.001	<0.001	<0.001
BMI (kg/m^2^)	23.2 (21.8–24.1)	38.9 (37.4–44.8)	34.4 (30.1–39.3)	<0.001	<0.001	<0.001
Body fat (%)	26.0 (23.8–29.9)	49.9 (47.3–51.8)	46.8 (40.0–5.03)	0.005	0.03	0.009
Liver volume (L)	1.3 (1.2–1.4)	2.3 (2.1–2.3)	1.9 (1.6–2.1)	<0.001	0.012	<0.001
HbA1c (mmol/mol)	33.2 (32.3–35.2)	40.5 (37.8–42.8)	36.5 (34.0–37.8)	<0.001	0.251	0.013
Fasting glucose (mM)	5.0 (4.7–5.2)	7.1 (6.4–7.4)	5.4 (5.1–6.2)	<0.001	0.096	<0.001
Fasting insulin (U/L)	3.0 (2.0–6.0)	23.5 (15.5–28.5)	11.0 (8.0–13.5)	<0.001	0.007	0.002
Basal ISR (pmol/min/m^2^)	63.9 (54.1–76.9)	141 (124–167)	108 (99.5–147)	<0.001	0.002	0.070
VLDL-TAG (mM)	0.35 (0.30–0.57)	0.77 (0.66–1.04)	0.68 (0.60–0.86)	0.041	0.116	0.505
2-h OGIS (mL/min/m^2^)	451 (429–481)	306 (272–358)	365 (337–382)	<0.001	0.004	0.067
HOMA_IR_ (fraction)	0.9 (0.7–1.3)	4.6 (4.2–7.9)	2.7 (2.0–4.1)	<0.001	0.006	0.001
Glucose sensitivity (pmol/min/m^2^/mM)	75.9 (63.1–91.8)	49.4 (36.2–59.4)	69.5 (59.0–89.6)	0.241	0.999	0.048
Rate sensitivity (pmol/m^2^/mM)	546 (411–938)	523 (337–807)	796 (621–1014)	0.871	0.763	0.392
Insulin clearance (L/min/m^2^)	2.8 (2.2–3.2)	1.3 (1.1–1.5)	1.7 (1.6–2.2)	0.002	0.166	0.004

Data are presented as median (IQR).

*P for obese patients pre-surgery vs controls; ^†^*P* for obese patients post-surgery vs controls; ^‡^*P* for obese patients post- vs pre-surgery.

HbA1c, glycated hemoglobin; HOMA_IR_, homeostatic model assessment of insulin resistance; ISR, insulin secretion rate; OGIS, oral glucose insulin sensitivity index; VLDL-TAG, very-low density lipoprotein-triacylglycerol.

### Glucose, pancreatic and gut hormones during the mixed-meal test

**Figure 3:**
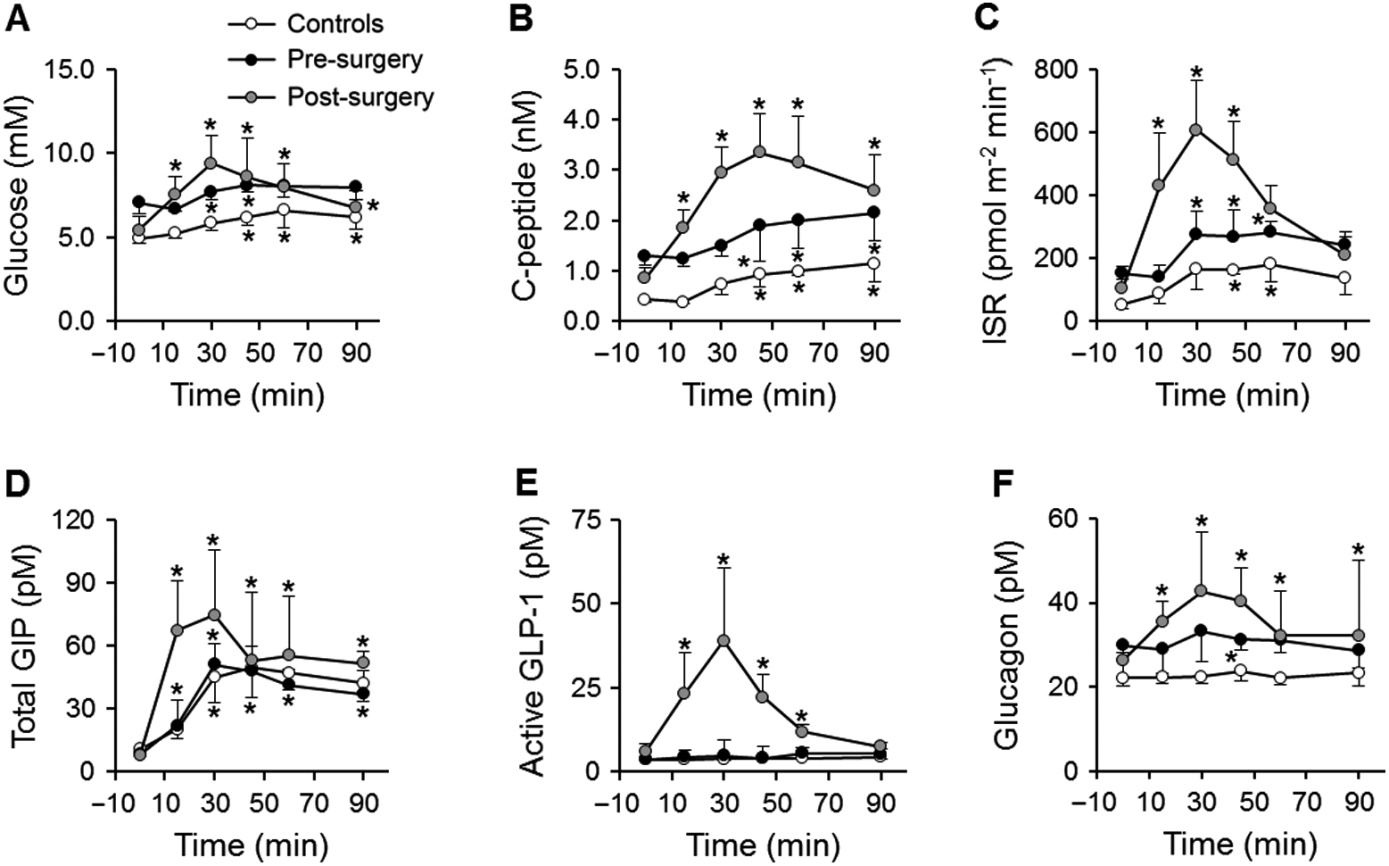
Plasma glucose (A), C-peptide (B), insulin secretion rate (ISR) (C), total GIP (D), active GLP-1 (E) and glucagon (F) during the mixed-meal test in controls (white balls) and subjects before (black balls) and after bariatric surgery (grey balls). Data are presented as median (IQR). *P < 0.05 vs baseline in linear mixed model with Tukey–Kramer’s correction.

Incremental glucose response to a mixed-meal was similar in obese subjects and controls (Fig. 3A). After surgery, fasting and 2-h glucose were decreased, although incremental glucose response was markedly larger and the post-prandial peak in plasma glucose levels occurred earlier than before surgery. A similar pattern was observed for C-peptide and for ISR (Fig. 3B and C). Consequently, insulin clearance was lower during the mixed-meal test than at baseline in all groups (*P* < 0.001). Plasma GIP increased similarly in all groups, whereas plasma GLP-1 levels were increased only in subjects after having undergone surgery (Fig. 3D and E). Plasma glucagon levels were unchanged in controls and increased in obese subjects, especially after surgery (Fig. 3F).

### Bariatric surgery enhances PV flow response to a mixed meal

**Figure 4:**
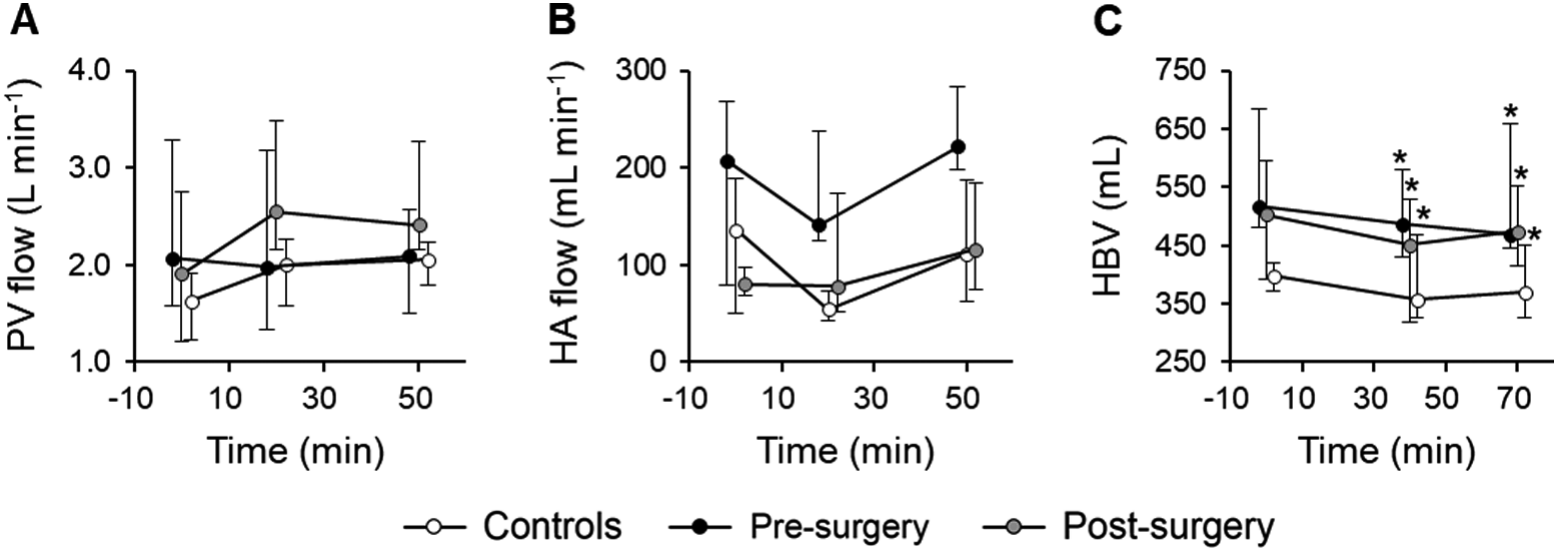
Portal vein flow (A), hepatic artery flow (B), and hepatic blood volume (C) responses to a mixed-meal in controls (white balls) and subjects before (black balls) and after bariatric surgery (grey balls). Values are expressed as blood flow or volume per whole liver. The relative contributions of portal vein and hepatic artery to the total hepatic blood flow were approximately 90 and 10%, respectively. Data are presented as median (IQR). Note the difference in time axis. *P < 0.05 vs baseline in linear mixed model with Tukey–Kramer’s correction.

Basal blood flow in PV and HA were similar in obese subjects before surgery and controls, while HBV was higher (516 (482–684) vs 398 (373–420) mL, *P* = 0.046) in the former group due to difference in liver volume. Mixed-meal did not alter hepatic blood flow at 20- and 50-min post ingestion, whereas HBV was slightly reduced (by 10.3 (3.5–15.0) %, *P* < 0.001) in both obese subjects before surgery and controls (Fig. 4A, B and C). Bariatric surgery decreased basal blood flow in HA (*P* = 0.046 compared to pre-surgery value) without affecting blood flow in PV or HBV (both NS). The effect of mixed-meal on HBV (reduction) and blood flow in HA (no change) was similar after than before surgery. In contrast, blood flow in PV increased rapidly by 1.65-fold from baseline (*P* = 0.026) in both post-surgical groups (Supplementary Fig. 1A and B); however, VSG-treated subjects tended to have steeper increase in PV blood flow post-prandially than subjects in the RYGB group.

### HBV response to a mixed-meal is reproduced by GIP infusion

**Figure 5:**
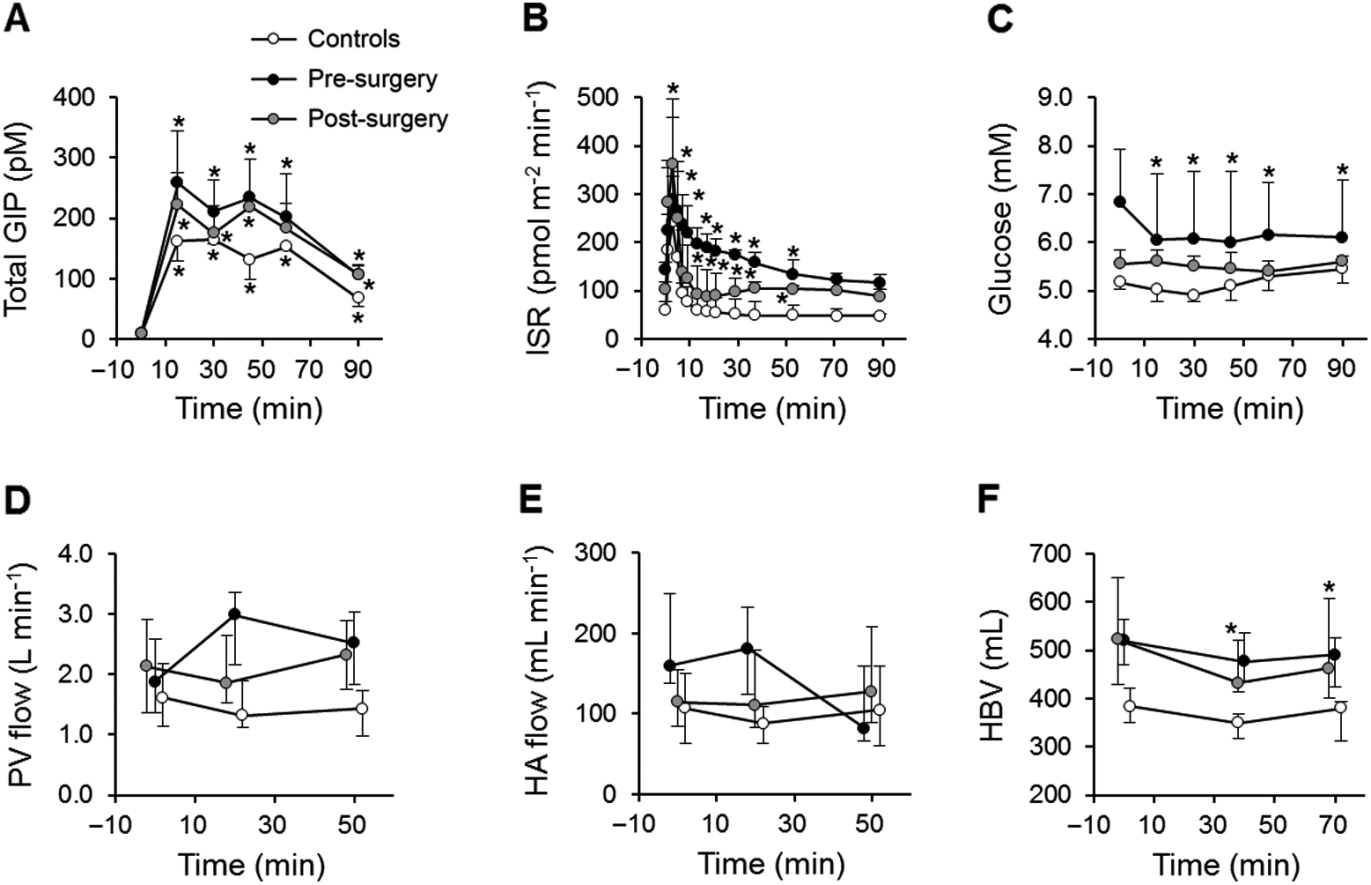
Plasma total GIP (A), insulin secretion rate (ISR) (B), glucose (C), and hepatic blood dynamic parameters (D, E and F) during GIP infusion in controls (white balls) and subjects before (black balls) and after bariatric surgery (grey balls). Data are presented as median (IQR). Note the difference in time axis. *P < 0.05 vs baseline in linear mixed model with Tukey–Kramer’s correction.

During the GIP infusion, supra-physiological GIP levels (17) were achieved in all groups, and this was accompanied by an increase in ISR (Fig. 5A and B) and glucagon (*P* < 0.001). Plasma glucose decreased in subjects before surgery but remained unchanged in controls and subjects after surgery (Fig. 5C). Insulin clearance decreased by 0.2 (0.0–0.5) L/min/m^2^ (*P* = 0.002) from baseline during the infusion in all groups. GIP affected neither PV nor HA flow (Fig. 5D and E) in any group; HBV was decreased in all subjects after surgery by a median of 89.1 (61.3–119) mL (*P* < 0.001), but it was less pronounced and nonsignificant in controls and subjects before surgery (Fig. 5F). Pancreatic or gut blood flow responses were not associated with HBV response in any group.

### An opposite effect of GLP-1 was seen on liver blood flow in lean controls

**Figure 6:**
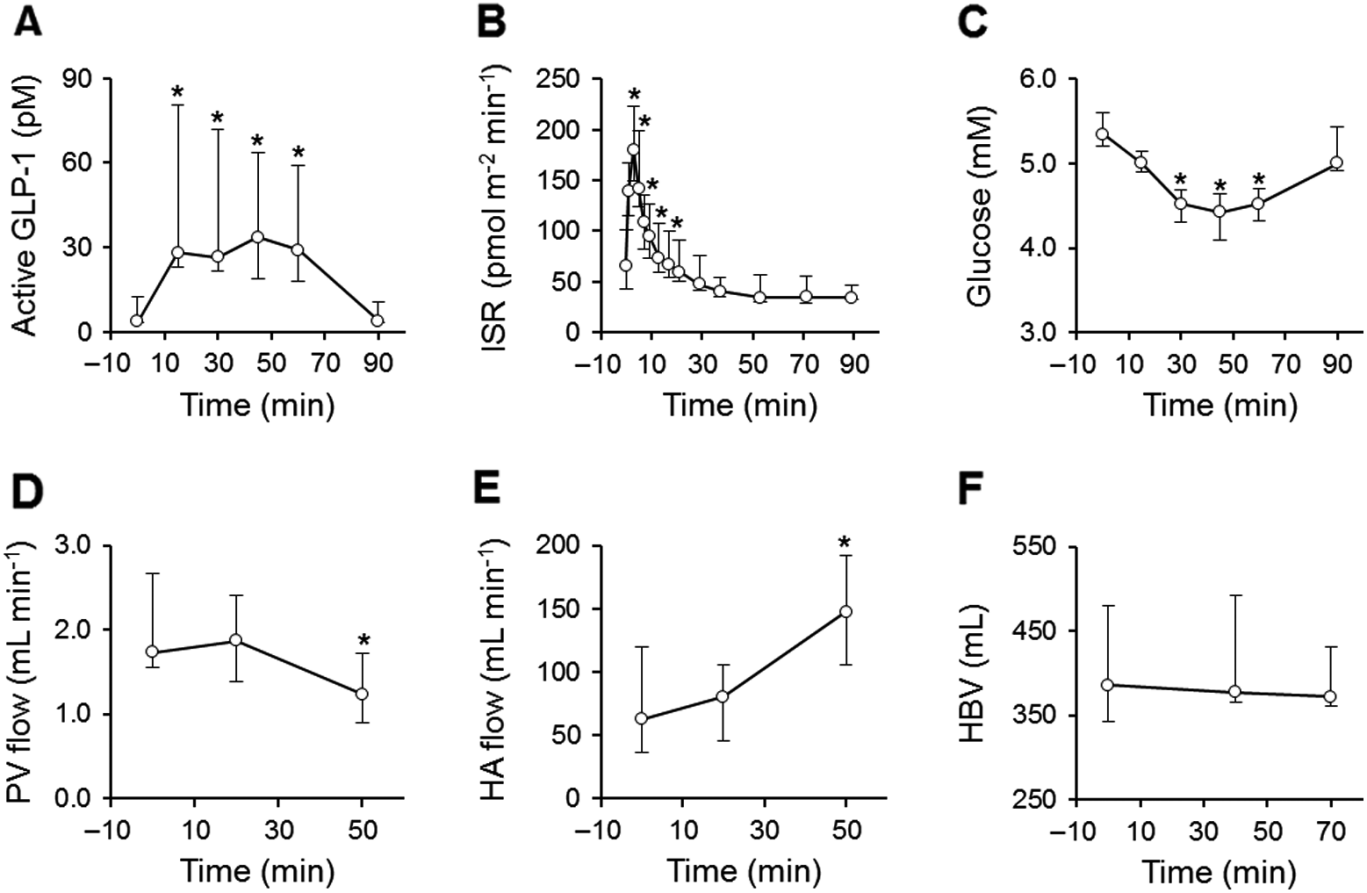
Plasma active GLP-1 (A), insulin secretion rate (ISR) (B), glucose (C), and hepatic blood dynamic parameters (D, E and F) during GLP-1 infusion in controls. Data are presented as median (IQR). Note the difference in time axis. *P < 0.05 vs baseline in linear mixed model with Tukey–Kramer’s correction.

Active GLP-1 levels were raised to post-prandial levels in all controls, although the response was clearly augmented in three subjects (Fig. 6A and Supplementary Fig. 2). ISR was increased early after the onset of infusion and decreased thereafter to sub-basal levels, and glucose was decreased by 1.0 (0.7–1.3) mM despite basal normoglycemia (Fig. 6B and C). PV flow decreased whereas HA flow increased late after the start of infusion (Fig. 6D and E). In contrast, HBV was stable throughout the experiment (Fig. 6F).

## Discussion

Bariatric surgery leads to rapid gastric emptying, enhanced effect of incretin hormones and reduced liver fat content (18, 19, 20, 21, 22), whereas little is known about splanchnic blood dynamics after surgical gastrointestinal anatomical change. To shed some light on these issues, we utilized PET/MRI technology to quantify hepatic blood perfusion and volume during a mixed-meal test in morbidly obese subjects with T2D before and after bariatric surgery, as well as in lean non-diabetic controls.

The most salient finding of our study was that PV flow was enhanced post-prandially only in subjects after bariatric surgery without significant difference between RYGB and VSG groups. This result is in line with our previous report (7) showing markedly increased blood flow in the gastrointestinal tissues after bariatric surgery. Even though both of these surgeries lead to increased gastric emptying rate and comparable hormonal responses to meal ingestion (19), VSG lacks the foregut exclusion (of RYGB) and leaves the pyloric sphincter intact; therefore, the dynamics of glycemic response to meal and mechanisms of weight loss/diabetes remission differ between the two bariatric procedures. This was evidenced by the steeper rise in PV flow in VSG-treated subjects of the present cohort, which is likely the result of the jejunal hyperemia in this group (7). Collectively, these data suggest that rapid gastric emptying and intestinal nutrient transit elicit a general stimulation in splanchnic circulation after bariatric surgery, possibly resulting in improved nutrient absorption and post-prandial glucose tolerance.

The role of incretins in the regulation of splanchnic blood flow is not well characterized. Previous studies in dogs have shown that frequent GIP injections lead to a dose-dependent increase in PV flow and decrease in HA flow, respectively, whereas GLP-1 administration induces vasodilation in rat mesenteric arteries (23, 24). In the present study, we showed that supraphysiologic GLP-1 levels during fasting state actually decrease PV flow in lean controls, similarly as in pancreas (5). This finding is in concert with a study by Trahair *et al*. (25), who observed a modest reduction in superior mesenteric artery (SMA) flow in healthy older fasted subjects. As pancreatic islet flow is largely dependent on plasma glucose levels (26), we hypothesize that the decrease in splanchnic flow observed during GLP-1 administration is caused by hypoglycemia rather than GLP-1 directly. This is supported by the fact that the reduction in PV flow in our data was observed only when glucose levels had fallen significantly when compared with baseline. On the contrary, GIP infusion did not affect PV or HA flow in any of the studied groups. In our previous report (7), we showed that GIP administration increases gut flow and decreases pancreatic flow, suggesting that these opposite vascular effects are compensated for in the liver.

Post-prandial blood drainage to extrahepatic sites, such as skeletal muscle, may improve peripheral glucose uptake and reduce glucose excursions (27). Here, we showed that a mixed-meal decreased HBV to a similar extent in all groups demonstrating a preserved liver blood reservoir function in morbidly obese subjects with no changes after bariatric surgery. We also show that GIP causes a rapid decrease in HBV similar to that observed during the mixed-meal test, independent of the changes in gastrointestinal blood flow. While the reduced HBV response to GIP infusion was most pronounced in obese subjects after bariatric surgery, there was a tendency towards a decrease in all groups. Given that fractional hepatic extraction of GIP is minimal (28), it is likely that the reduction in HBV during incretin infusion is secondary to the redistribution of blood between gastrointestinal (gut and pancreas) and peripheral tissues.

GIP acts as a physiological blood glucose stabilizer associated with stimulation of insulin and glucagon secretion during hyper- and hypoglycemia, respectively, in healthy subjects and in patients with T2D (10, 29). Despite transient upregulation of insulin secretion by GIP infusion in all groups, plasma glucose was decreased to a normoglycemic range only in (hyperglycemic) subjects before surgery, suggesting preserved GIP action in the pancreatic islets of T2D subjects.

The strengths of our study include the use of PET and validated models to estimate hepatic blood dynamics, providing detailed information *in vivo* in humans. Additionally, our study subjects were demographically similar to previous studies (19, 20) investigating the effects of bariatric surgery on whole-body metabolism in subjects with T2D. Conversely, we appreciate that this study had a few limitations: (1) Our sample size was rather small and consisted of surgical subjects who had heterogenous operations (although there was no significant difference between surgical groups). Furthermore, BMI-matched controls were excluded to gain insight on the physiological regulation of splanchnic blood flow in normal-weighted individuals. (2) The infusion provoked nearly three-fold higher GIP levels than what was seen during the mixed-meal test. (3) In the lack of magnetic resonance spectroscopy or liver biopsies, it was not possible to quantitate hepatic fat content. However, in another study from our group (30), liver fat content was decreased by 76% six months after bariatric surgery in similar subjects as in the present study. These data suggest that a decrease in hepatic fat may have occurred in our subjects in response to bariatric surgery. (4) GLP-1 was not infused in obese subjects due to radiation dose limits.

In conclusion, we have shown that blood flow response in portal vein to a mixed-meal is enhanced and GLP-1 secretion is stimulated early after bariatric surgery in morbidly obese subjects with T2D, suggesting that the altered gastrointestinal anatomy in the post-bariatric state improves digestion and nutrient absorption also by influencing splanchnic blood flow. On the other hand, HBV response to a mixed-meal is influenced by GIP thereby diverting blood to extrahepatic sites. These data would suggest that incretins are important regulators of splanchnic metabolism and blood dynamics, with effects extending beyond the pancreatic islets.

## Supplementary Material

Supporting Figure 1

Supporting Figure 2

Supporting Table 1

## Declaration of interest

The authors declare that there is no conflict of interest that could be perceived as prejudicing the impartiality of the research reported.

## Funding

This study was conducted within the Finnish Centre of Excellence in Molecular Imaging in Cardiovascular and Metabolic Research and was supported by the Academy of Finland and Finnish Cultural Foundation.

## Author contribution statement

H H and J K contributed to the design of the study, acquired and researched data, and wrote the manuscript. S K was responsible for the surgical procedures, contributed to the design of the study and discussion, and edited the manuscript. N K calculated portal vein and hepatic artery flow rates, offered technical support, and edited the manuscript. S H was responsible for the statistical analyses, and edited the manuscript. A M and R P researched data, contributed to the discussion, and edited the manuscript. A L, N W, L G, and P N contributed to the design of the study and discussion, and edited the manuscript. All authors approved the final version of the manuscript.
