# Supplementary figures and images for: Liver blood dynamics after bariatric surgery: the effects of mixed-meal test and incretin infusions
Source: Endocr Connect. 2018 Jun 25;7(7):888–96. doi: 10.1530/EC-18-0234 (PMC6063878; doi:10.1530/EC-18-0234)

**A**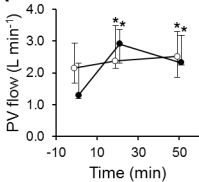**B**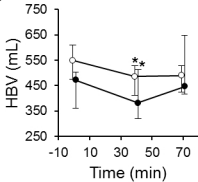

—○— RYGB —●— VSG

Supplement: Supporting Figure 1 [file ec-7-888-s001.pdf]

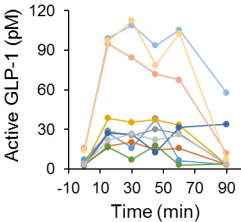

Supplement: Supporting Figure 2 [file ec-7-888-s002.pdf]
