# Supplementary material for: Liver blood dynamics after bariatric surgery: the effects of mixed-meal test and incretin infusions
Source: Endocr Connect. 2018 Jun 25;7(7):888–96. doi: 10.1530/EC-18-0234 (PMC6063878; doi:10.1530/EC-18-0234)
Supplement: Supporting Table 1 [file ec-7-888-t001.pdf]

SUPPLEMENTARY TABLES

Supplementary Table 1. Outcomes of surgery between RYGB and VSG groups. *P* for surgery factor (RYGB *vs.* VSG) and time × surgery interaction was greater than 0.05 for all parameters.

	RYGB		VSG	
	Pre-surgery	2-months	Pre-surgery	2-months
n (male/female)	5 (2/3)		5 (0/5)	
Weight (kg)	121 (120-131)	104 (102-111)*	102 (93.2-126)	83.0 (80.6-114)*
BMI (kg m^{-2})	38.4 (38.3-40.0)	33.0 (31.2-37.1)*	39.4 (34.7-46.8)	35.7 (29.8-42.7)*
Body fat (%)	49.9 (30.4-50.3)	46.8 (23.6-46.8)*	49.7 (47.5-52.2)	46.9 (42.6-50.7)*
HbA1c (mmol mol^{-1})	41.0 (36.0-43.2)	34.0 (34.0-36.0)*	40.0 (40.0-42.1)	37.0 (37.0-38.0)*
Fasting glucose (mM)	7.0 (5.5-7.5)	5.6 (5.3-5.7)*	6.8 (5.6-6.9)	5.9 (5.6-5.9)*
Fasting insulin (mU L^{-1})	22.0 (17.0-28.0)	11.0 (7.0-12.0)*	14.0 (13.0-19.0)	10.0 (8.0-18.0)*
Fasting GIP (pM)	13.5 (12.9-13.9)	8.7 (6.5-10.4)	10.8 (5.5-11.8)	8.9 (5.3-19.7)
Fasting GLP-1 (pM)	8.7 (3.2-8.8)	7.2 (6.4-7.9)	4.7 (3.6-6.6)	4.6 (4.2-6.4)
2-hour OGIS (mL min^{-1} m^{-2})	264 (240-297)	385 (329-423)	313 (313-374)	361 (358-369)
$HOMA_{IR}$ (fraction)	6.8 (4.2-9.7)	2.8 (1.6-3.0)*	4.6 (4.2-4.7)	2.6 (2.1-4.5)*
Glucose sensitivity (pmol min^{-1} m^{-2} mM^{-1})	50.4 (50.3-62.4)	95.6 (68.6-148)	34.9 (33.8-48.5)	68.3 (55.8-70.4)
Rate sensitivity (pmol m^{-2} mM^{-1})	483 (407-859)	937 (804-1718)	562 (314-650)	595 (504-701)
Insulin clearance (L min^{-1} m^{-2})	1.2 (1.1-1.3)	1.7 (1.6-2.3)*	1.4 (1.3-1.6)	1.7 (1.4-2.0)*

Data are presented as median (IQR). *$P < 0.05$ for time factor. GIP, glucose-dependent insulinotropic polypeptide; GLP-1, glucagon-like peptide 1; HbA1c, glycated hemoglobin; $HOMA_{IR}$, homeostatic model assessment of insulin resistance; OGIS, oral glucose insulin sensitivity index.
